# Clustering Algorithms on Low-Power and High-Performance Devices for Edge Computing Environments

**DOI:** 10.3390/s21165395

**Published:** 2021-08-10

**Authors:** Marco Lapegna, Walter Balzano, Norbert Meyer, Diego Romano

**Affiliations:** 1 Department of Mathematics and Applications, University of Naples Federico II, 80126 Napoli, Italy; 2 Department of Electrical Engineering and Information Technology, University of Naples Federico II, 80126 Napoli, Italy; walter.balzano@unina.it; 3 Poznan Supercomputing and Networking Center, 61139 Poznan, Poland; meyer@man.poznan.pl; 4 Institute for High Performance Computing and Networking, National Research Council, 80131 Napoli, Italy; diego.romano@icar.cnr.it

**Keywords:** edge computing, low-power devices, multi-core computing, GPU computing, clustering algorithms

## Abstract

The synergy between Artificial Intelligence and the Edge Computing paradigm promises to transfer decision-making processes to the periphery of sensor networks without the involvement of central data servers. For this reason, we recently witnessed an impetuous development of devices that integrate sensors and computing resources in a single board to process data directly on the collection place. Due to the particular context where they are used, the main feature of these boards is the reduced energy consumption, even if they do not exhibit absolute computing powers comparable to modern high-end CPUs. Among the most popular Artificial Intelligence techniques, clustering algorithms are practical tools for discovering correlations or affinities within data collected in large datasets, but a parallel implementation is an essential requirement because of their high computational cost. Therefore, in the present work, we investigate how to implement clustering algorithms on parallel and low-energy devices for edge computing environments. In particular, we present the experiments related to two devices with different features: the quad-core UDOO X86 Advanced+ board and the GPU-based NVIDIA Jetson Nano board, evaluating them from the performance and the energy consumption points of view. The experiments show that they realize a more favorable trade-off between these two requirements than other high-end computing devices.

## 1. Introduction

The extensive use of devices aimed to collect data through sensor networks according to the Internet of Things (IoT) paradigm produces large datasets representing the knowledge base for addressing significant problems of the modern society [1,2]. As examples of applications based on pervasive data collections, it is possible to report: (i) smartphones or other devices to monitor and predict traffic in large cities to reduce pollution and oil consumption [3]; (ii) environmental sensors on leisure yachts and charter vessels to forecasting the spatial and temporal presence of pollutants in the proximity of mussel farm [4]; (iii) microwave sensors mounted on drones for SAR data processing to monitoring biophysical parameters in extended geographical regions [5]. In all these cases, the data can be sent to centralized servers to define global analysis and forecasts according to a model known as *cloud computing* [6], implemented even through virtualization techniques [7,8].

The main problem of this approach is that such datasets require, on the one hand, the presence of communication networks with low latency and large bandwidth, and on the other hand, the use of large-scale high-performance computing systems. An alternative to this centralized model is based on a distributed data processing, directly carried out by small computing devices closely connected to the sensors, thus defining a model known as *edge computing* [9]. An intermediate model of distributed architecture is known as *fog computing* where multiple edge devices operate under the supervision of a local server in a Local Area Network [10].

We see several benefits in using this paradigm. First of all, the data preprocessing near the collection place allows a reduction in the volume of information to be transmitted to company servers, saving on the cost of communication networks. Furthermore, the described model appears to be more fault-tolerant, as a single failed device can be easily isolated from the network without interrupting the overall service and replaced at a low cost. Moreover, perhaps even through the consortia of several companies, a distributed data collection model allows greater democratization and open access to knowledge. A further advantage of this model is associated with the privacy and security of data because it is possible to significantly reduce sensitive information from spreading on the network. Finally, it allows time-sensitive applications to free themselves from dependence on central servers, improving the real-time potential.

For such reasons, in the current years, we have been observing the rising of a further specialized model identified as High-Performance Edge Computing (HPEC). In this scenario, the needs of high-performance computing move from large and expensive data centers toward small, low-power, high-performance tools deployable on the edge of networks, capable of running complex compute-intensive applications. Consequently, several devices have been introduced on the consumer market that closely integrates, on the same board, microcontrollers for the sensors management and computing units with architectures based on multi-core CPU or GPU, implementing various and sophisticated forms of parallelism. In this regard, we point out that the adoption of parallel architectures inside the computing systems is precisely one of the main strategies to enhance performance without increasing energy consumption [11].

Some of the application areas of most significant interest for edge computing are, without doubt, Artificial Intelligence (AI) and Machine Learning (ML). As an example of this fruitful connection, several authors distinguish between *AI for edge computing*, aimed to improve the efficiency and effectiveness of the infrastructure through AI techniques, and *AI on edge*, aimed to shift intelligence and decision-making ability to the edge of the network, through AI techniques. A comprehensive work, deepening the relations between AI and ML from one side and edge computing from the other one, is [12].

Among the most used ML methodologies, clustering algorithms play a central role in gathering similar data in the same group according to a precise metric. Many surveys focus on clustering techniques and often offer several distinct views and classifications of the algorithms. However, all of them emphasize the computational peculiarities of the *K*-means algorithm, featured by simplicity, ability to scale with the number of attributes and elements, and a reasonable computational complexity mainly in advanced computing systems. For such a reason, the *K*-means algorithm is probably one of the most studied procedures in the ML field [13,14,15].

Several efforts have been addressed toward parallel implementations of the *K*-means algorithm in several high-performance computing environments in the last years. Significant algorithms are described, for example, in [16] for distributed memory architectures, in [17,18,19] for multi-core CPUs and in [20] for GPUs based systems. Almost all these studies emphasize the role of a large amount of data as a critical feature to enable an implementation based on the data parallelism programming model.

However, to our knowledge, few studies have been conducted to implement and evaluate clustering algorithms developed explicitly for high-performance and low-power devices. One of them is [21] where the authors compare the performance of three distinct algorithms on an NVIDIA Jetson Xavier platform. In [22] the authors introduce a two-level learning model where the lower level trains a series of local models on multiple edge nodes with limited storage and computing resource, whereas the upper level computes a global model by averaging the trained gradients of each client. However, it should be noted that these two works reenter more correctly in the fog computing field because of the powerful hardware resources used in the experiments and the multilevel software structure. Another paper assessing the problem of moving machine learning applications on low-power devices is [23] where the authors design a novel strategy to optimize the performance of deep learning applications on edge nodes. Finally, in [24], the authors present an FPGA system-on-chip-based architecture that supports the acceleration of ML algorithms in an edge computing environment.

Usually, the process of taking real-time decisions at the edge of the network comprises two distinct steps: (i) data collection through sensors and their organization in some data structure; (ii) real-time analysis of data near the collection place to take suitable actions. Because the latency for the data streaming between the two stages can be considered negligible in the boards for high-performance edge computing, the present work focuses on the second step that, eventually, can represent the bottleneck of the entire process. With more detail, the contribution of this paper is three-fold, addressing some issues not thoroughly investigated in the previous papers: (1) we discuss two distinct forms of parallelism exploitable in a clustering algorithm, based on the features of the boards used for the experiments: a quad-core UDOO X86 Advanced+ board and a GPU-based NVIDIA Jetson Nano board; (2) we improve an existing clustering algorithm for multi-core CPUs for enabling its execution on GPU-based devices, leveraging the previous parallelism models; (3) we introduce a new model based on several parameters combining performance and energy consumption to evaluate the efficiency and effectiveness of the algorithms in edge computing environments.

The paper is then structured as follows: in Section 2 we design a parallel Adaptive *K*-means Algorithm for devices with different architectures; in Section 3 we describe a model for an edge computing environment and the devices we have used for the experiments; in Section 4 we carried out experiments with four different datasets to stress the algorithm in various possible scenarios, the results of which we discuss in Section 5; we report final considerations and ideas for future works in Section 6.

## 2. Parallelization Strategies for the Adaptive *K*-Means Algorithm

This section has several objectives: first, we recall the Adaptive *K*-means Algorithm introduced in [18,25] which dynamically increases the number of clusters until reaching a satisfactory homogeneity of the elements in each group. Then, we give some implementation details relating to the employed data structures, intending to describe the different forms of parallelisms introduced in them, taking into account the underlying computing devices’ architecture.

### 2.1. The Adaptive K-Means Algorithm

The Basic *K*-means Algorithm acts on a dataset $S=\{x_{n}:\;x_{n}\in R^{d},\;\;n=1,...,N\}$ containing *N* elements of the *d*-dimensional space. Given an integer *K*, its main aim is to organize the items $x_{n}\in S$ in *K* non empty subsets $C_{k}$ (called *clusters*) making up a partition $P_{K}=\left\{C_{k}:C_{k}\subseteq S,\;\;k=1,...,K\right\}$ of the dataset *S*. Each cluster contains $N_{k}$ elements grouped according a given similarity criterion and it is identified with a *d*-dimensional representative vector $c_{k}$ called centroid, defined by means of the vector operation:

(1) $$c_{k}=\frac{1}{N_{k}}\sum_{x_{n}\in C_{k}}x_{n}\;\;\;\;\;k=1,...,K$$

Usually, the homogeneity among two elements is measured through the Euclidean distance in the *d*-dimensional space, so that the *K*-means algorithm iteratively reorganizes the partition $P_{K}$ reassigning each element $x_{n}$ to the cluster $C_{\delta }$ minimizing the Euclidean distance from its centroid $c_{\delta }$, that is:

(2) $$\left|\right|x_{n}-c_{\delta }{\left|\right|}_{2}=min_{k=1,...,K}\left|\right|x_{n}-c_{k}{\left|\right|}_{2}$$

until there are not changes in the partition (or the number of the reassignments can be considered negligible).

The following Algorithm 1 is a description of the Basic *K*-means.
**Algorithm 1:** Basic *K*-means Algorithm(1) Initialize *K* clusters $C_{k}$ with $N_{k}$ elements $x_{n}\in S$ (2) **repeat**     (2.1) **for** each cluster $C_{k}\in P$: update clusters info      (2.2) **for** each $x_{n}\in S$: search the cluster $C_{\delta }$ as in (2)      (2.3) **for** each $x_{n}\in S$: displace $x_{n}$ in $C_{\delta }$   **until** (none (or negligible) changes in the reassignment)


It is important to note that, in real problems, it is often impossible to define the value of *K* as input data. For such reason, in [18,25] we have introduced an Adaptive *K*-means Algorithm which increases the value of *K* iteratively until a quality parameter meets the user’s requirements. Among the large number of indices proposed in the literature, we used the Root-Mean-Square standard deviation (RMSSD) [13]:

(3) $$R_{P_{K}}={\left[\frac{\sum_{k=1}^{K}\sum_{x_{n}\in C_{k}}\left|\right|s_{n}-c_{k}{\left|\right|}_{2}^{2}}{d(N-K)}\right]}^{1/2}$$

The RMSSD calculates the average affinity of elements in clusters $C_{k}$ so that the algorithm can increase *K* until the (3) does not show significant reduction when adding a new cluster. The above strategy used to define the number of clusters is known as the “Elbow” method [26].

We observe that in an iterative procedure that increases the number of clusters, at step *K* the partition $P_{K-1}$ already groups the element in *K* clusters according to their similarity. Thus, to reduce the computational cost related mainly to the displacement of the elements among the clusters, our algorithm operates only on the elements showing still little affinity. Usually, the standard deviation

(4) $$V_{k}=\sqrt{\frac{1}{N_{k}-1}\sum_{n=1}^{N_{k}}\left|\right|x_{n}-c_{k}{\left|\right|}_{2}^{2}}$$

measures the affinity of a set of $N_{K}$ elements in a cluster: the smaller the value $V_{k}$ is, more similar the elements $x_{n}$ with the centroid $c_{k}$ are. Therefore, at each step *K*, our algorithm splits into two subclusters $C_{\alpha }$ and $C_{\beta }$ only the cluster $C_{\gamma }\in P_{K-1}$ with the largest standard deviation, that is:

(5) $$C_{\gamma }\;\;\;\mathrm{such that}\;\;\;V_{\gamma }=max_{k=1,...,K-1}\;V_{k}$$

and defines the new partition $P_{K}$ by means:

(6) $$\begin{array}{c} K=0\;\;\;\;\;P_{0}=\left\{C_{0}\right\}\;\;\;\mathrm{where}\;\;\;C_{0}\equiv S\\ K\geq 1\;\;\;\;\;P_{K}=P_{K-1}-\left\{C_{\gamma }\right\}\cup \left\{C_{\alpha },C_{\beta }\right\} \end{array}$$

With the previous definition at hand, we present the following Algorithm 2 as a description of the Adaptive *K*-means Algorithm (see [18,25] for further details):

We remark that the previous ideas take inspiration from well known and widely used procedures known as adaptive algorithms, which operate only on subproblems that show poor values of some quality index, such as, for example, the discretization error in the case of the computation of integrals or computational fluid dynamics (e.g., [27,28,29,30]).

**Algorithm 2:** Adaptive K-means Algorithm(1) Set the number of clusters K=0
(2) **repeat**
     (2.1) Increase the number of clusters K=K+1     (2.2) find the cluster $C_{\gamma }$ as in (5)     (2.3) define the new partition of clusters $P_{K}$ as in (6)     (2.4) **repeat**               (2.4.1) **for** each cluster $C_{k}\in P$: update clusters info               (2.4.2) **for** each $x_{n}\in S$: search the cluster $C_{\delta }$ as in (2)                (2.4.3) **for** each $x_{n}\in S$: displace $x_{n}$ to $C_{\delta }$      **until** (no change in the reassignment)     (2.5) update $R_{P_{K}}$ as in (3) 
   **until** (the variation of $R_{P_{K}}$ is smaller than a given threshold) 


### 2.2. Data Structures Organization

Clustering algorithms deal with large amounts of data that need to be stored in very efficient data structures where the whole organization and the data access strategies are critical points in achieving high performance. These issues are even more critical in low-power devices characterized by limited available memory and high latency.

**Figure 1:**
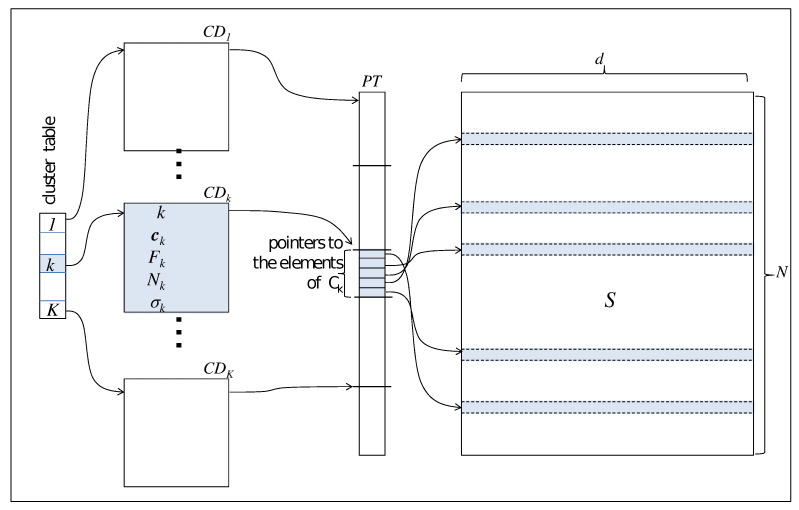
Organization of the main data structures in the parallel Adaptive *K*-means Algorithm.

Referring to Figure 1, each *d*-dimensional element $x_{n}$ of the dataset is stored in a row of a 2-dimensional N×d static array *S*. This implementation choice ensures better performance than others using dynamic data structures. Furthermore, since moving each element between clusters (step 2.4.3 of Algorithm 2) has a non-negligible cost of O(d) accesses to memory with a significant impact on the performance, our algorithm leaves the physical storage of the rows unchanged.

For accessing the elements in the array *S*, the algorithm then uses a pointers array PT, where contiguous items point to the elements of the same cluster. With this structure at hand, Algorithm 2, in step 2.4.3, can displace the elements only acting on the array PT with a cost of O(1) memory accesses.

All information featuring a cluster $C_{k}$ are stored in a structure $CD_{k}$ called Cluster Descriptor, containing:

- *k*: the cluster identifier
- $c_{k}$: the centroids of the cluster
- $F_{k}$: a pointer to the item of PT referencing the first cluster element
- $N_{k}$: the number elements in the cluster
- $V_{k}$: the standard deviation of the elements of the cluster

The Cluster Descriptor is updated in step 2.4.1 of Algorithm 2.

The last table of pointers CT, called Cluster Table, provides direct access to the Cluster Descriptors.

### 2.3. Forms of Parallelism

Nowadays, the standard architecture model of the primary high-performance computing systems is organized around computing nodes based on multi-core CPUs with arithmetic accelerator devices [31]. For such a reason, a mandatory first step in building next-generation software stacks for edge computing platforms should be an efficient implementation of clustering algorithms for devices with similar features.

A multi-core CPU contains several independent computing units sharing resources on the node like main memory and bus. To reduce the bottleneck in accessing such resources, they own multiple levels of private cache memory. Furthermore, a private set of registers allows the operating system to dispatch independent threads, enabling the implementation of algorithms designed according to the shared memory Multiple Instruction Multiple Data (MIMD) programming model. The main problem concerning these devices is the risk of *race condition* that is, the achievement of nondeterministic results depending on the thread scheduling order on shared elements in the main memory. For such reason, tools like the semaphores allow the definition of code sections that threads access one at a time.

A floating-point accelerator implements an entirely different parallel programming model. It is based on an architecture initially designed to accelerate typical operations in the images processing field such as texture mapping, rendering, and manipulations of figures’ pixels in a coordinate system. In any case, nowadays, high-level programming environments allow their use also for some general-purpose applications. Such devices possess thousands of elementary computing cores operating under common control, implementing a special form of Single Instruction Multiple Data (SIMD) vector processing. Due to this organization, they are well suited to high-throughput computations that exhibit strong data-parallelism. For such a reason, they support traditional CPUs in the sections of the applications requiring massive vector operations, where they can reach several orders of magnitude higher performance than a conventional CPU.

An analysis of the data structures described in Figure 1 allows us to recognize multiple forms of parallelism in the Adaptive *K*-means Algorithm, mainly inside the iteration structure 2.4) representing the computational kernel of the whole procedure. This study allows to achieve the high-performance features in an edge computing environment, where the sensors boards are designed around a hybrid architecture with multi-core CPUs and floating-point accelerators:

step 2.4.1. This step updates the critical features of each cluster stored in the cluster descriptors $CD_{k}$, such as the centroid (1) and the standard deviation of its items (4). Since the updating are independent of each other, the number of clusters *K* in the cluster table *CT* is the first level of parallelism with medium granularity we can introduce in the algorithm. We can call it *parallelism at cluster level*. With a multi-core CPU, the operating system can then schedule independent threads, updating the *K* clusters descriptors $CD_{k}$ in a parallel section.
step 2.4.2. In this task, the total number of elements *N* in the data structure *S* determines a finer degree of parallelism, and two strategies can be recognized. Firstly, it is possible to design a multithread version of this step where each thread manages N/P elements independently, with *P* the number of computing units. Furthermore, mainly with a significant value of *N*, the SIMD programming model, characteristic of a floating-point accelerator device, is especially suited for handling the independent items $x_{n}$ in this section of the algorithm. In both cases, we can call these strategies *parallelism at item level*.
step 2.4.3. This step represents a critical section in Algorithm 2. More precisely, in this step, each element $x_{n}$ can be eventually assigned to a new cluster according to its distance from the centroid, as in (2). This step can be the source of a high risk of race condition because different threads access the pointer array *PT* concurrently. Step 2.4.3 is then the only task managed in a sequential form in our Adaptive *K*-means Algorithm. On the other hand, this task is a data-intensive step of the whole algorithm.

From the above, we can say that a clustering algorithm like Algorithm 2 is a suitable benchmark to test several features of the devices for an edge computing architecture because it contains both compute-intensive steps (2.4.1 and 2.4.2) and a data-intensive step (2.4.3). Furthermore, it allows testing multiple parallel computing models, like the MIMD shared-memory model and the SIMD model for GPU computing.

## 3. The Edge Computing Platforms

This section presents a model for an edge computing architecture and introduces the low-power and high-performance devices we used to assess the Adaptive *K*-means Algorithm in this environment.

### 3.1. A Model for Edge Computing Architectures

**Figure 2:**
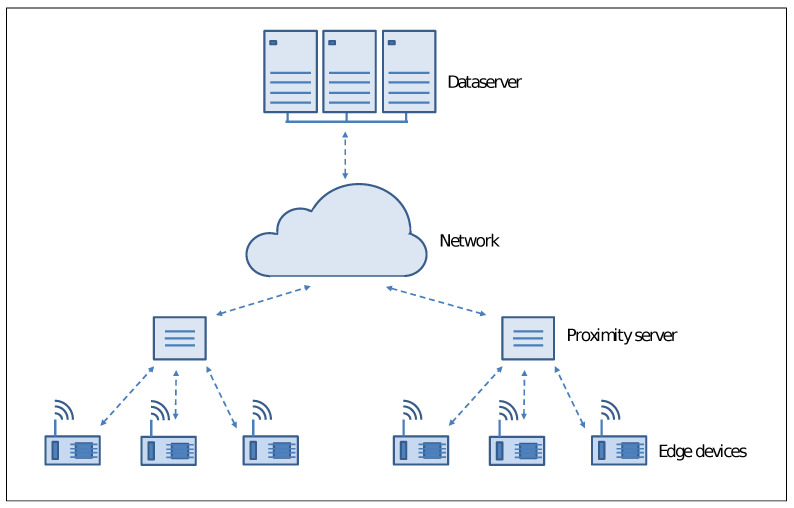
The basic edge computing architecture.

Following Figure 2 illustrates the basic structure of edge computing environment that we can describe as a multilayer architecture.

Peripheral computing resources connected with the sensors deployed at the network’s edge represent the lowest layer of the architecture. They allow close interaction with the user running real-time applications with prompt responsiveness and high quality of services. In any case, they have a limited capacity of computing power and storage, so that for some applications, it is necessary to forward data or specific requests to the upper level of the architecture.

At the intermediate level, there are proximity servers aimed to support devices at the edge. Several activities can be carried out at this level: from data caching to load balancing among devices to data transfer without completely involving the network. The proximity of these resources to the network peripheral allows better performance with a limited increase in latency.

Centralized high-end computing resources like high-performance clusters and large data servers compose the highest level of the architecture, where the primary aim is to receive data and information from the underlying level periodically. Since these computing resources reside far from the edge devices, they exhibit significant latency, mainly for large environments. In any case, they can provide supplementary computing resources in terms of data processing and storage to run large applications and build global models.

It is easy to see that the peripheral structure of an edge computing architecture is a critical issue. In the event of a massive number of resource-constrained devices requiring significant compute support from proximity servers, there is a high risk of losing the benefits of low latency and high responsiveness, which remains one of the primary goals of such software architecture. On the other hand, the availability of devices with sophisticated computing resources, even if small in size and with low energy consumption, avoid frequent request to the proximity server, guaranteeing the best Quality of Service level.

### 3.2. UDOO X86 Advanced+

The UDOO X86 Advanced+ [32] (2017) is a 120 × 85 mm board integrating an Intel Celeron N3160 (Braswell) CPU with 4 core running at 2.24 GHz and an Intel Curie microcontroller implementing the Arduino 101 interface. A 4GB dual-channel DDR3L Memory RAM is directly soldered on-board, while a 32 GB eMMC acts as secondary memory. The board features a total of 36-pin GPIO available on the external Pinout connectors. Furthermore, other standard interfaces are available (1 HDMI and two miniDB++ ports, 4 USB and 1 microSD ports, 1 Bluetooth module, 1 Gigabit Ethernet interface, 1 slot for a wireless module, and 1 HD audio connector). The board needs to be supplied with an external 12 V power unit with a declared Thermal Design Point (TDP) of 6 Watt. In the following, for our experiments, we use the TDP as an estimate of the maximum energy consumption per second of a CPU when running real applications. The operating temperature ranges between 0 and 60 degrees Celsius. We provided the board with a Linux Ubuntu 18.04 distribution with a standard GNU C compiler and POSIX thread library.

### 3.3. NVIDIA Jetson Nano

The NVIDIA Jetson Nano [33] (2019) is a small computing board (only 69 × 45 mm) mounting a stripped-down version of the System-on-Chip Tegra X1. It needs to be supplied with an external 5 V power unit with a declared TDP set to 5 or 10 Watt. The board integrates an ARM Cortex A57 CPU with 4 core running at 1.47 GHz (0.918 GHz when working at 5 Watt) and an NVIDIA Maxwell GPU with 128 core running at 921 MHz (640 MHz when working at 5 Watt). A 4 GB 64-bit LPDDR4 chip is available on the board as main memory, while a slot can accommodate a microSD card as secondary memory. The board mounts 40-pin GPIOs, and several standard interfaces with the outside world are available (4 × USB, 1 × HDMI, 1 × Gigabit Ethernet, M.2 Key E for Wifi support). The operating temperature ranges between −25 and 80 Celsius. The Jetson Nano Developer Kit that can be downloaded from NVidia provides a Linux Ubuntu operating system and the main components of the CUDA environment.

## 4. Experiments

This section describes the experiments we conducted to verify the effectiveness of the low-power devices in running the Adaptive *K*-mean algorithm. First, we introduce the test problems, and then we report the results both from the performance and the energy consumption point of view.

### 4.1. Test Problems

We assessed the effectiveness and efficiency of the Adaptive *K*-means Algorithm on the described low-power devices on some classic datasets taken from the University of California (UCI) Machine Learning Repository [34]. The datasets represent cases coming from different application areas, and different values feature them for the number of items *N*, the dimension *d*, and the number of clusters *K*, to consider several scenarios:

**Letters**  **[35].** This problem is a middle-size dataset where the aim is to identify a black-and-white rectangular image as a letter in the English alphabet. The whole dataset contains N= 20,000 images, each of them representing a character through d=16 numerical attributes describing some specific feature (dimension of the image, edge counts, number of pixels, ...). For this test, the number of clusters is K=26.
**Wines**  **[36].** In this problem, the items represent N=4898 samples of Portuguese wines through d=11 numerical attributes describing some organoleptic features (e.g., acidity, sweetness, alcohol content). According to their quality, the wines are clustered in K=11 groups (a value from 0 to 10).
**Cardio**  **[37].** In this problem, the dataset items represent N=2126 fetal cardiotocographic reports (CTGs), each of them described with d=21 diagnostic features (e.g., acceleration, pulsation, short and long term variability, and other physiological features). The primary aim of the dataset is to classify the elements in K=10 morphologic patterns.
**Clients**  **[38].** In this dataset, the items refer to a marketing campaign of a banking institution. Each of the N= 45,211 elements is the numerical description of possible clients according to d=16 features (e.g., job, education, age, marital status). The items are classified in K=2 clusters.

### 4.2. Tests on the UDOO X86 Advanced+ Board

To test Algorithm 2 on the UDOO X86 Advanced+ board, we implemented it with the standard C language in the Ubuntu Linux 18.04 operating system; the POSIX Thread library has been used to handle the threads implementing the parallelism at cluster level in step 2.4.1, and the parallelism at the item level in step 2.4.2. More precisely, in step 2.4.1, the *K* clusters are assigned to the *P* threads in a round-robin fashion operating on the cluster table *CT*. Similarly, in step 2.4.2, the *N* items are assigned to the *P* threads in chunks of N/P contiguous elements, operating on the pointers array *PT*.

The purpose of the first experiment is to evaluate the performance of the Adaptive *K*-means Algorithm using the described test cases through the traditional values of Speed-up and Efficiency:

$$S_{P}=\frac{T_{1}}{T_{P}}\;\;\;\;\;\;\;E_{P}=\frac{S_{P}}{P}$$

In the previous definitions, $T_{P}$ is the total execution time with *P* threads. We observe here that, on each core of the modern CPUs, it is generally possible to run several threads, but, in such a configuration, they share computation time alternating use ofphysical resources without effective parallel execution. For this reason, we run the tests with only one thread per core in order to measure the effective concurrency of the compute units.

**Figure 3:**
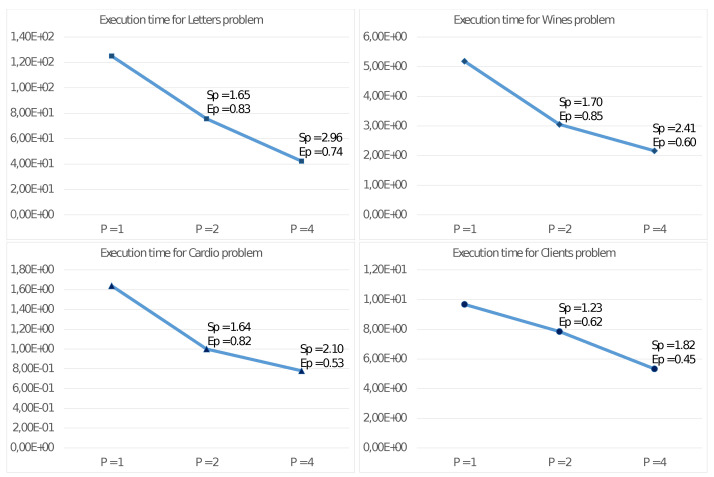
Execution time (in seconds), Speed-up and Efficiency of Algorithm 2 on the UDOO X86 Advanced+ board.

Figure 3 reports the results of such experiments on the described test problems, showing the execution time in seconds, the Speed-up, and the Efficiency with P=1, P=2, and P=4 threads.

**Figure 4:**
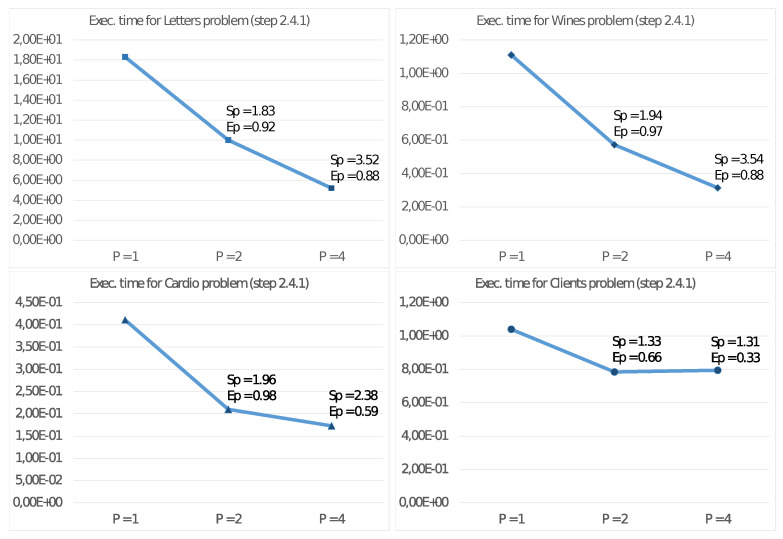
Execution time (in seconds), Speed-up and Efficiency of step 2.4.1 Algorithm 2 on the UDOO X86 Advanced+ board.

**Figure 5:**
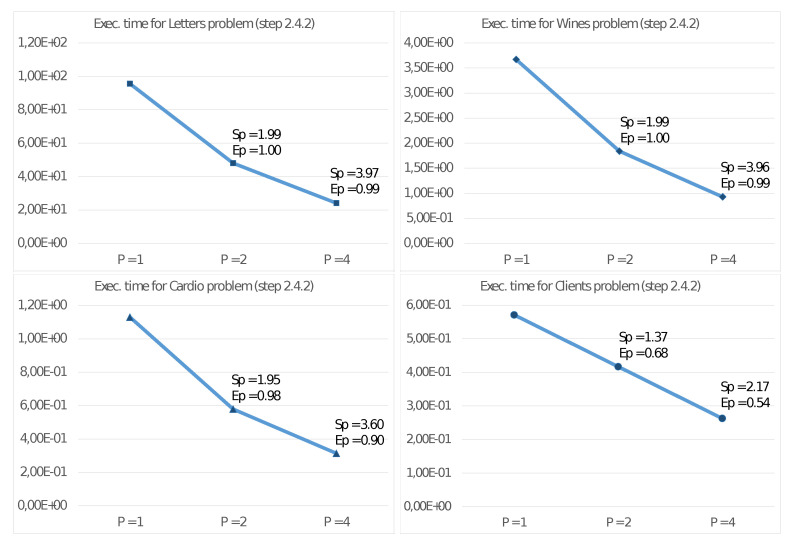
Execution time (in seconds), Speed-up and Efficiency of step 2.4.2 of Algorithm 2 on the UDOO X86 Advanced+ board.

Furthermore, we report in Figure 4 and Figure 5 the execution times and corresponding values for $S_{P}$ and $E_{P}$ also for steps 2.4.1 and 2.4.2 of the algorithm. With these values, we can then estimate the effective parallelism implemented in these steps. In this regard, we remind that step 2.4.3 is a sequential task and does not significantly benefit from increasing the number of threads.

Finally, for a more penetrating evaluation of the UDOO X86 Advanced+ board when running Algorithm 2, let us compare its energy consumption with the two following systems:

**Haswell-DT:**  (H-DT) a 4-core Intel Core i5-4460S CPU for desktop segment running at 2.9 GHz with a TDP of 65 Watt (2014).
**Coffee Lake-S:**  (CL-S) an 8-core Intel Core i9-9900K CPU for server segment running at 3.6 GHz with a TDP of 95 Watt (2018).

To estimate the energy consumption of a given CPU, we consider the product among the execution time (in second) and the TDP (in Joule/second) More precisely:

(7) $$H^{\left(CPU\right)}=T_{4}^{\left(CPU\right)}\cdot TDP^{\left(CPU\right)}=\left[second\right]\cdot \frac{\left[Joule\right]}{\left[second\right]}$$

where we introduced the CPU name as a superscript. From the dimensional point of view, the (7) is an estimate of the energy consumed by a given CPU for executing Algorithm 2 with P=4 threads. With this definition at hand, we then introduce the following ratio:

$$R_{1}=H^{\left(CPU\right)}/H^{\left(UDOO\right)}$$

**Figure 6:**
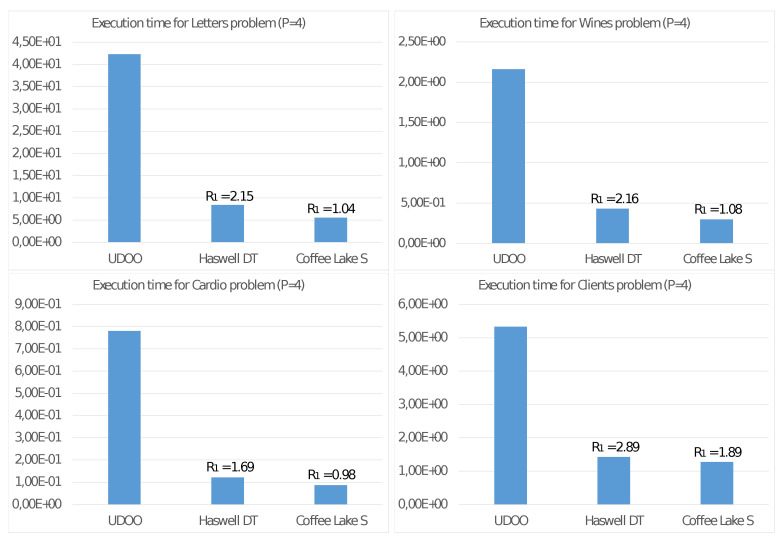
Execution time (in seconds) and energy consumption ratio $R_{1}$ of two high-end CPU compared with the UDOO X86 Advanced+ board.

The value of $R_{1}$ estimates how more significant the energy consumption $H^{\left(CPU\right)}$ is than that of the equivalent value $H^{\left(UDOO\right)}$ of the UDOO board used as a baseline. In Figure 6 we report the execution time of Algorithm 2 on the UDOO X86 Advanced+ board with P=4 threads compared with that of the two systems based on the Haswell-DT and the Coffe Lake-S CPUs. In the same figure, we also report the values of $R_{1}$. Finally, we remark that we conducted these experiments always using P=4 core. For such reason, we then consider only half of the TDB of the Coffee Lake-S CPU to consider the reduced number of computing units we employed.

### 4.3. Tests on the NVIDIA Jetson Nano Board

To test Algorithm 2 on the NVIDIA Jetson Nano board, we realized a new implementation with the standard C language in the Ubuntu Linux 18.04 operating system supplied with the native Developer Kit. The POSIX Thread library has been used to parallelize steps 2.4.1 and 2.4.2, as described for the UDOO X86 Advanced+ board code. Furthermore, we employed the CUDA programming environment to introduce parallelism at the item level in step 2.4.2. More precisely, in this step, the N/P items assigned to each POSIX thread are handled in a CUDA kernel with the same number of independent threads. With this strategy, each CUDA thread is in charge of executing step 2.4.2 on a single item that can be accessed through the pointers array *PT*.

**Figure 7:**
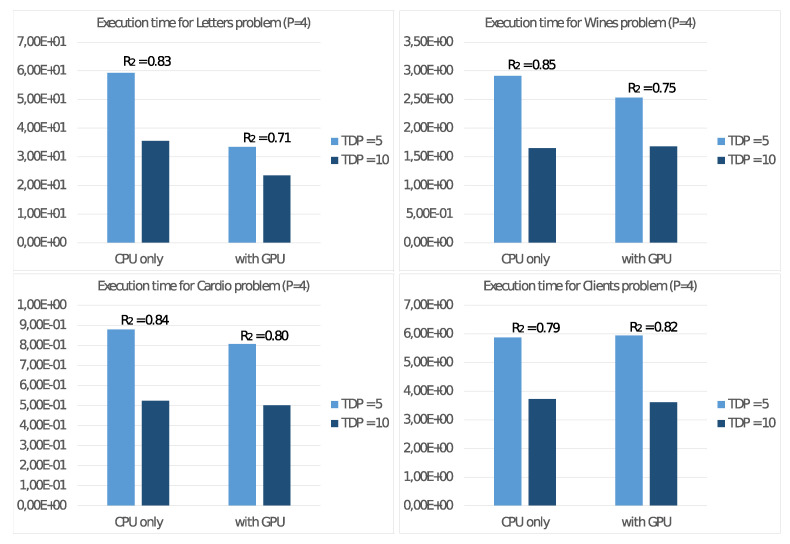
Execution time (in seconds) and energy consumption ratio $R_{2}$ of the NVIDIA Jetson Nano with two different TDP.

In the first experiment, we evaluate the energy efficiency with the board set at 5 and 10 Watt. To this aim, in a similar way to the (7), we consider the energy consumption with P=4 threads $H^{\left(TDP\right)}=T_{4}^{\left(TDP\right)}\cdot TDP$ using the device TDP setting as superscript. With this definition, we report the total execution times in seconds $T_{4}^{\left(TDP\right)}$ with the two settings in Figure 7, where we introduced also the values of the ratio:

$$R_{2}=H^{\left(5\right)}/H^{\left(10\right)}$$

that measures the power consumption of the 5 Watt setting versus the 10 Watt setting. The values refer to the execution with only CPU usage as well as with the GPU usage to accelerate floating-point computation.

In all cases, the ratio $R_{2}=H^{\left(5\right)}/H^{\left(10\right)}$ is always less than 1, showing a correlation between the frequency setting and the energy consumption. For such reason, the subsequent experiments on the NVIDIA Jetson Nano board are always run with the 5 Watt setting.

**Figure 8:**
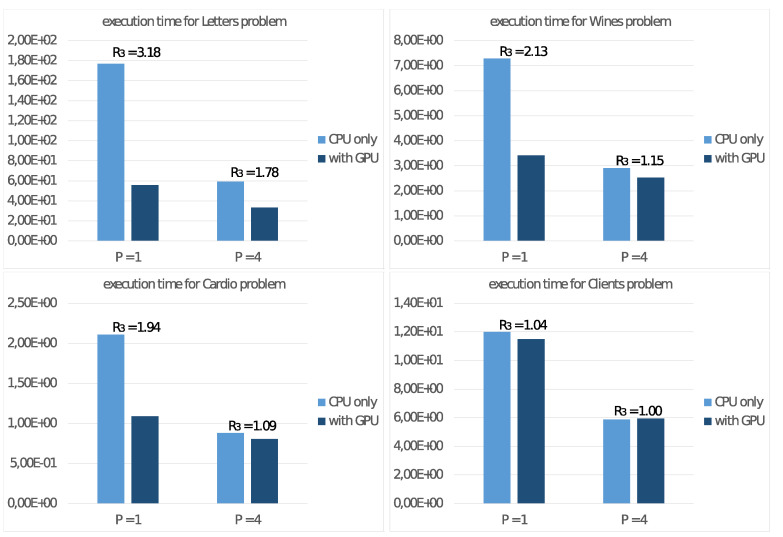
Execution time (in seconds) of Algorithm 2 on the NVIDIA Jetson Nano board and performance gains $R_{3}$ obtained through GPU usage.

To study the performance of the NVIDIA Jetson Nano board on the test cases described at the beginning of this section, let us consider the execution time $T_{P}$ achieved running Algorithm 2 only on the host CPU with P=4 threads. Then we consider the execution time $T_{P}^{*}$ achieved with the use of the GPU as a floating-point accelerator, and then we calculate the ratio

$$R_{3}=T_{P}/T_{P}^{*}$$

that represents the performance gain obtained through GPU usage. Following Figure 8 reports the achieved values with P=1 and P=4 threads on the CPU and the performance gain $R_{3}$.

**Figure 9:**
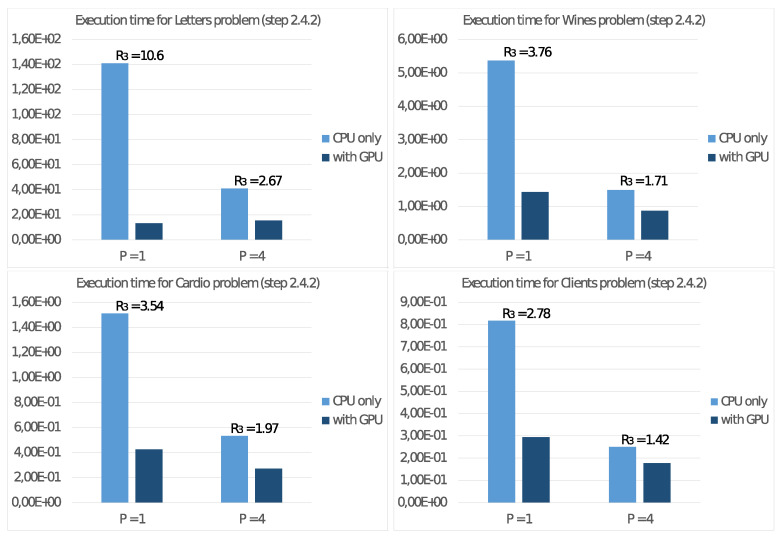
Execution time (in seconds) of step 2.4.2 of Algorithm 2 on the NVIDIA Jetson Nano board and performance gains $R_{3}$ obtained through GPU usage.

Furthermore, we report in Figure 9 the same values only for step 2.4.2 of the algorithm. With these values, we can then estimate the effective parallelism implemented in this step. In this regard, we remind that step 2.4.2 is the only task implementing the SIMD programming model, characteristic of a floating-point accelerator device.

**Figure 10:**
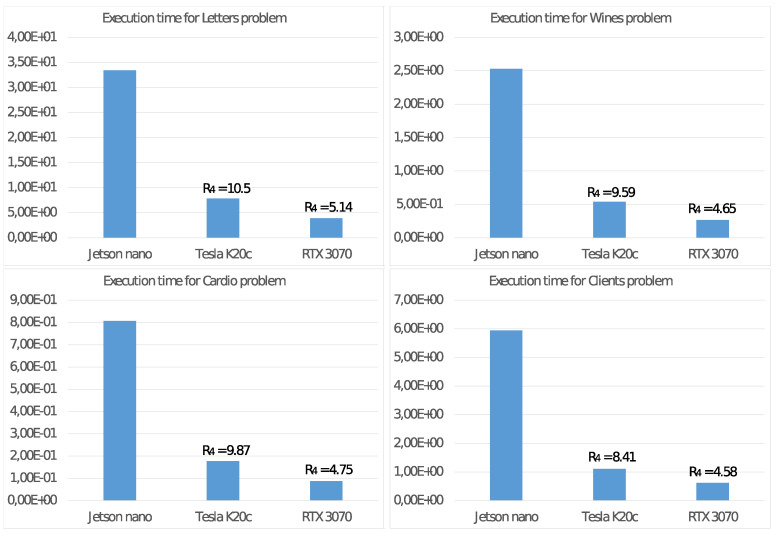
Execution time (in second) and energy consumption ratio $R_{4}$ of two other GPUs compared with the NVIDIA Jetson Nano board.

We complete the analysis of the features of the NVIDIA Jetson Nano, comparing the energy consumption with those of two other systems mounting a GPU as a floating-point accelerator:

**Tesla K20c:**  (T-K20) a system based on an NVIDIA Tesla K20c GPU (2012) with 2496 CUDA cores running at 0.706 GHz with a global memory of 5 GBytes and a TDP of 225 Watt. The host CPU is a 4-core Intel Core i7-950 running at 3.07 GHz;
**RTX 3070:**  (RTX) a system based on an NVIDIA GeForce RTX 3070 GPU (2020) with 5888 CUDA cores running at 1.75 GHz with a global memory of 8 GBytes and a TDP of 220 Watt. The host CPU is an 8-core Intel Core i9-9900K running at 3.6 GHz;

For these two systems, defined $H^{\left(GPU\right)}$ similarly as in (7), in Figure 10, we report the execution time of Algorithm 2 by using the GPU as a floating-point accelerator for the CPU when running with P=4 threads and the values of the ratio:

$$R_{4}=H^{\left(GPU\right)}/H^{\left(Jetson\right)}$$

that evaluates how greater the energy consumption $H^{\left(GPU\right)}$ is than that of the equivalent value $H^{\left(Jetson\right)}$ used as a baseline.

## 5. Discussion

Figure 3 shows good values for the Speed-up $S_{P}$ and Efficiency $E_{P}$ with the quad-core UDOO X86 Advanced+ board. Mainly for significant problems (e.g., Letter), we obtain very high values also with four threads. This achievement is even more evident from observing the results reported in Figure 4 and Figure 5, showing the performance only for steps 2.4.1 and 2.4.2 able to exploit the MIMD shared memory parallelism of the CPU. Step 2.4.1 implements parallelism among the cluster, so the efficiency values increase with *K*. As an example, for the Client problem, where K=2, we achieve unsatisfactory values. Figure 5 shows even better results for $S_{P}$ and $E_{P}$, where the parallelism is implemented among the *N* elements $x_{n}$, allowing a better load balancing among the threads. In any case, a crucial issue for devices of an edge computing environment is their ability to achieve high performance with a reduced energy consumption per unit time. Figure 6 allows us to study this fundamental aspect. Comparing the execution times of two high-end multi-core CPUs, with those of the UDOO X86 Advanced+ board, we observe that, with P=4 threads, the Haswell-DT and the Coffe Lake-S CPUs executed Algorithm 2 between four and eight times faster, but with a TDP it was about ten times more significant. The ratio $R_{1}$ among the energy consumptions $H^{\left(CPU\right)}=T_{4}^{\left(CPU\right)}\cdot TDP^{\left(CPU\right)}$ for the execution of Algorithm 2 show that, from this point of view, the UDOO X86 Advanced+ board is as efficient as the Coffe Lake-S CPU and between two and three times more efficient than the Haswell-DT CPU.

It is possible to draw similar conclusions looking at the results of the experiments on the NVIDIA Jetson Nano board. First of all, we already noted in Figure 7 that the best energy efficiency is achieved with the TDP set to 5 Watt. From the performance point of view, Figure 8 shows a significant performance increment when using a GPU as a floating-point accelerator, mainly for significant problems (e.g., Letter problem), confirming the devices’ ability to carry out real ML applications. These conclusions are more evident by observing the values in Figure 9, where are reported the execution time and the performance gain $R_{3}$ for step 2.4.2 of the Adaptive *K*-means Algorithm, implementing suitable parallelism for the GPU. For low-dimension problems, namely the Cardio problem (with N=2126 items in the dataset) or the Clients problem (with only K=2 clusters), we report a less significant performance gain because such values are not able to exploit the computational power of the GPU. Much more interesting are the tests aimed to measure energy consumption. The comparison of the execution times on the NVIDIA Jetson Nano $T_{4}^{*}$ and on the other systems $T_{4}^{\left(GPU\right)}$ in Figure 10 shows that the NVIDIA Jetson Nano board executes the Adaptive *K*-means Algorithm from 5 to 10 times slower than the NVIDIA Tesla K20c and NVIDIA RTX 3070 cards, but with much smaller power consumption. More precisely, the achieved values of $R_{4}=H^{\left(GPU\right)}/H^{\left(Jetson\right)}$ show that the NVIDIA Jetson Nano board is between 4 and 10 times more energy-efficient than other high-level GPUs.

The results achieved from the experiments confirm, therefore, the possibility of the low-power and high-performance devices being used effectively in an edge computing environment because of their ability to achieve good performance with reduced energy consumption on actual clustering algorithms. In both cases, the best energy efficiency is achieved at the expense of a lack of absolute performance, sometimes necessary in some real-time processes. However, it is interesting to point out that the low economic cost of these devices (between 100 and 200 Euros on the consumer market) allows, if necessary, to quickly increase the number of data collection and processing points, implementing a form of distributed computing which eventually involves only the proximity servers responsible for load balancing. This strategy is remarkably flexible and efficient, as it allows to increase the aggregate computational power only in the network sections with this need, without using more powerful devices that require more energy.

## 6. Conclusions

In the last ten years, we have witnessed an exponential growth of sensor networks that collect data in various application areas to transmit them to remote servers for subsequent processing. However, the Internet of Things model thus created produces enormous volumes of data every day that require, on the one hand, networks with low latency and high bandwidth, and on the other hand, high-performance computing resources. Therefore, it is often preferable to directly process the data at the collection site through low-energy devices that integrate sensors with the computing resources on the same card.

In this work, we concentrated our attention on the implementation and evaluation of algorithms on some small-size devices that promise to provide high-performance computing power with reduced energy consumption. As a case study, we used a clustering algorithm that has the characteristics to be helpful to support decisions on the edge of the network without the involvement of central servers. The algorithm has been implemented in two versions to consider the different architectures used and tested using data sets with different features to consider several possible scenarios.

The obtained results show that these current devices for high-performance computing for edge computing, while not reaching absolute computing powers comparable to modern high-end CPUs, exhibit a better ratio between the achieved performance and energy consumption when executing clustering algorithms. The synergy of such devices and Machine Learning algorithms confirms the premise of widespread deployment of decision-making points in the network’s periphery and opens new scenarios in several scientific and social fields.

In any case, fundamental problems still need to be solved, and further work is required before we see widespread and efficient networks with distributed decision points. Among these, it remains to define the strategies for balancing and scheduling the tasks between peripheral devices and centralized servers with the associated coordination protocols, the development of machine learning applications capable of processing streaming of data collected in real-time by the sensors, or the issues related to the delivery of a federated global identity management framework for provisioning secure ICT services, or the development of mixed precision algorithms to improve the ratio between performance and energy consumption. Some of these aspects will be the subject of further works still in progress that can benefit from methodologies developed for similar scenarios with distributed structure and high-performance demand even in wireless networks [39,40,41].

## Data Availability

Not applicable.
